# Measuring Vulnerability in Grief: The Psychometric Properties of the Italian Adult Attitude to Grief Scale

**DOI:** 10.3390/ejihpe13060074

**Published:** 2023-06-04

**Authors:** Alessio Gori, Eleonora Topino, Pierluigi Imperatore, Alessandro Musetti, Julius Sim, Linda Machin

**Affiliations:** 1 Department of Health Sciences, University of Florence, Via di San Salvi 12, Pad. 26, 50135 Florence, Italy; 2 Integrated Psychodynamic Psychotherapy Institute (IPPI), Via Ricasoli 32, 50122 Florence, Italy; imperatore.pierluigi@gmail.com; 3 Department of Human Sciences, LUMSA University of Rome, Via della Traspontina 21, 00193 Rome, Italy; eleonora.topino@gmail.com; 4 Department of Humanities, Social Sciences and Cultural Industries, University of Parma, 43121 Parma, Italy; alessandro.musetti@unipr.it; 5 School of Medicine, Keele University, Staffordshire ST5 5BG, UK; j.sim@keele.ac.uk (J.S.); l.machin@keele.ac.uk (L.M.)

**Keywords:** adult attitude to grief scale, evaluation methods, grief, psychological testing

## Abstract

Although experiences of loss and the consequent grief are natural in human life, some individuals may have difficulty managing these events, to the point of developing significant impairment in their functioning in important life areas. Given this, the present research aimed to explore the psychometric properties of the Italian version of the Adult Attitude to Grief scale (AAG) to facilitate research on adult vulnerability to grief among Italian-speaking populations. A sample of 367 participants (*M_age_* = 30.44, *SD* = 11.21; 78% females) participated in this research. A back-translation procedure was implemented to develop the Italian AAG. Then, participants completed the Italian AAG alongside a battery of other self-report psychometric scales in order to assess aspects of the construct validity of the AAG: the Forty-Item Defense Style Questionnaire, the Impact of Event Scale—Revised, and the Beck Depression Inventory–II. A bifactor structure was found to have the best fit to the data, supporting the possibility of using both the general factor (i.e., vulnerability) and three dimensions (i.e., overwhelmed, controlled, and resilient). Unlike the original version, the control dimension emerged as a “protective” factor in the Italian population, together with the resilient factor. Furthermore, results provided satisfactory indications of internal consistency and construct validity. In conclusion, the Italian AAG was shown to be a valid, reliable, quick, and easy-to-use scale that can be used both for research and clinical practice in the Italian context.

## 1. Introduction

Grief can be defined as a “*dynamic, pervasive, highly individualized process of responding to a loss*” [1] (p. 2003), including, as a source of this state, not only the loss of a valued and beloved person, but also, for example, the loss of work, opportunity, or certain values [2]. Experiences of mourning and emotional pain related to loss are natural in human life as the result of processes of development, maturation, and changes that imply the loss of the status quo [3]. However, although there is a range of socially and culturally accepted pain responses considered normal following negative life events [4,5], some individuals may present pathological and maladaptive responses that may become chronic and persistent, leading to an intense condition of malaise that can significantly compromise their functioning in various areas of life [6,7,8]. In this regard, scientific literature has shown that problematic grief may be associated with both physical and mental illnesses. Indeed, populations with pathological responses to grief showed more cardiovascular problems [9], more infectious diseases [10], and increased mortality [11]. Concerning psychological outcomes, evidence has highlighted a greater risk of suicide [12], depression [13], anxiety [14], and PTSD [15]. Furthermore, problematic grief is also associated with interpersonal [16] and workplace [17] problems, as well as with lower levels of life satisfaction [18]. Given the aforementioned relevant clinical implications, due to the dysfunctional nature of this condition, the accurate evaluation of conditions of vulnerability to grief and, in association, the use and dissemination of effective screening tools for therapeutic and preventive applications appear to be of great importance.

In this regard, some valuable self-report measures have been described in the scientific literature. Among these, the Inventory of Complicated Grief (ICG) [19] helps clinicians assess indicators of pathological grief, such as anger, disbelief, and hallucinations; the PG-13-Revised (PG-13-R) [20] scale is a tool that offers a one-dimensional assessment of prolonged grief symptoms; the Brief Grief Questionnaire (BGQ) [21] is a short scale that aims to facilitate screening for complicated grief. Although these tools have demonstrated excellent psychometric properties and their usefulness in various contexts, they tend, predominantly, to consider grief related to the death of a loved person (excluding other dimensions of loss) and/or focus predominantly on negative dimensions, without also including the evaluation of personal inner resourcefulness that could be useful in the clinical setting. In this regard, the *Adult Attitude to Grief scale* (AAG) [22,23] emerged as a promising, theoretically oriented scale within this field that allows the assessment of vulnerability to mourning in its broadest conception while also allowing the evaluation of resilience responses, and it has already demonstrated utility in the clinical context [23].

### 1.1. The Adult Attitude to Grief Scale

The Adult Attitude to Grief scale (AAG) [22,23] is a self-report measure consisting of nine items, based on the theoretical foundation of the Range of Response to Loss (RRL) model [24,25]. In line with the last version of the model [25], the scale allows the user both to conduct an assessment of a general level of vulnerability in grief (the total score) and to outline a comprehensive profile of how responses to loss combine in dealing with this experience by considering three subscales:

1. The “overwhelmed” factor, indicating a feeling of being overcome with grief, being stressed, and having lost the meaning of life.
2. The “control” factor, indicating instinctive mechanisms to resist pain and maintain autonomy by avoiding full engagement with the loss.
3. The “resilient” factor, indicating the perception of having resources to cope with the grief and the hope of overcoming it.

In the original version [22,23], the combination of the overwhelmed, controlled, and the reverse of the resilient dimensions allowed for a general index of vulnerability in grief, conceptualized as a state of difficulty in dealing with loss and its consequences at emotional, social, and practical levels [22,23,25]. The scale showed good psychometric properties, with excellent indications of validity and reliability [23]. Furthermore, evidence highlighted valuable practical feedback on its use in different populations or contexts, such as adults following an expected death [26] or during the COVID-19 pandemic [27], supporting its utility in both research and therapeutic practice.

### 1.2. Aim and Hypothesis

Although the instrument has shown some useful implications for research and clinical practice, it has never been validated in an Italian population. Therefore, the present study aimed to translate and validate the Italian version of the AAG to facilitate research on adult vulnerability to grief among Italian-speaking populations. Given the satisfactory psychometric properties of the original instrument, it was hypothesized that the Italian version would have good reliability and high construct validity, and it would replicate the three factors that emerged in the original English version.

## 2. Materials and Methods

### 2.1. Participants

The research involved a sample of 367 participants (78% females) who reported having experienced loss in their life (see Table 1). Their ages ranged from 18 to 67 years (*M* = 30.44, *SD* = 11.206). Most (71%) were single, 34% had a university degree, 37% were students, and 23% were employees. Concerning the experience of loss, 60% reported having lived through experiences of mourning, 31% through separation from important people (e.g., friends, family, partner), and 9% through loss of work, job opportunities, or important occasions.

### 2.2. Procedure and Ethics

A back-translation procedure [28] was implemented to develop the Italian version of the AAG. During the first step, the items of the English version of the AAG [22,23] were translated into Italian by an independent translator. During the second step, the translated version was further back-translated into English. Finally, the outcome was discussed until the authors and the translators agreed on cross-language equivalence. The final version (see Appendix A) was administered online together with the other self-report measures, which were hosted on the Google Forms platform. Participants were recruited through a snowball method. Before starting, each participant was informed of the general aim of the research and provided informed consent electronically. All the procedures in this research were approved by the first author’s institutional Ethics Committee.

### 2.3. Measures

#### 2.3.1. Adult Attitude to Grief Scale (AAG)

The *Adult Attitude to Grief scale* (AAG) [22,23] is a 9-item self-report scale designed to assess vulnerability in grief. Items are rated on a 5-point Likert scale, from 0 (*strongly agree*) to 4 (*strongly disagree*), and both a total score (by summing all the items after reversing some specific ones; see the Results section for more information) and scores of three dimensions (overwhelmed, controlled, and resilient) can be calculated. The psychometric properties of the Italian AAG are presented in the Results section.

#### 2.3.2. Forty-Item Defense Style Questionnaire (DSQ-40)

The *Defense Style Questionnaire* (DSQ-40) [29,30] is a 40-item self-report questionnaire designed to assess defense mechanisms. Items are rated on a 9-point Likert scale, from 1 (*strongly disagree*) to 9 (*strongly agree*), and allow the evaluation of three dimensions: mature, neurotic, and immature defenses. The Italian version [30] was used in this research and showed acceptable internal consistency in the present sample (mature, 8 items, *α* = 0.67; neurotic, 8 items, *α* = 0.62; immature, 24 items, *α* = 0.84).

#### 2.3.3. Impact of Event Scale—Revised (IES-R)

The *Impact of Event Scale—Revised* (IES-R) [31,32] is a 22-item self-report questionnaire designed to assess post-traumatic stress symptoms. Items are rated on a 5-point adverbial scale, from 0 (*not at all*) to 4 (*extremely*), and both a total score and scores on three dimensions (intrusion, avoidance, and hyperarousal) can be calculated. The Italian version [32] was used in this research and showed acceptable internal consistency in the present sample (total score, *α* = 0.95; intrusion, 8 items, *α* = 0.81; avoidance, 8 items, *α* = 0.92; hyperarousal, 6 items, *α* = 0.89).

#### 2.3.4. Beck Depression Inventory-II (BDI-II)

The *Beck Depression Inventory-II* (BDI-II) [33,34,35] is a 21-item self-report questionnaire designed to assess depressive symptoms. Items are rated on a 4-point multiple-response scale ranging from 0 to 3, and both a total score and two dimensions (somatic/affective symptoms and cognitive symptoms) can be calculated. The Italian version [34,35] was used in this research and showed acceptable internal consistency in the present sample (total score, *α* = 0.93; somatic/affective symptoms, 12 items, *α* = 0.89; cognitive symptoms, 9 items, *α* = 0.89).

### 2.4. Data Analysis

Data were analyzed using SPSS (v. 21.0; IBM, New York, NY, USA) [36], AMOS (v. 24.0; IBM, New York, NY, USA) [37], and JASP (v. 0.17.2; JASP Team, Amsterdam, The Netherlands) [38] software for Windows. First, the normality of the data was explored by implementing item analysis, considering an absolute skew value ≤ 2 and an absolute kurtosis ≤ 7, as indicative of normality [39]. Next, the Kaiser–Meyer–Olkin (KMO) measure and Bartlett’s test of sphericity were implemented to investigate the suitability of the data for factor analysis: a KMO value > 0.7 and the statistical significance of the Bartlett’s test (*p* < 0.001) were considered as indicative of sampling adequacy for factor analysis [40]. Then, the factor structure of the Italian AAG was tested using confirmatory factor analyses (CFAs) and evaluation of the following fit indices: the discrepancy divided by degree of freedom (*χ*^2^/df), with values less than 5 suggesting a reasonable fit [41,42]; the Normed-Fit Index (NFI), the Tucker–Lewis index (TFI), and the Comparative Fit Index (CFI), where values > 0.90 suggest a reasonable fit [43,44,45]; and the Root Mean Square Error of Approximation (RMSEA) and Standardized Root Mean Square Residual (SRMR), suggesting a reasonable fit for values < 0.08 [46]. In addition, the Δ*χ*^2^ was used to compare the correlational factor structure that emerged from the original development study [22,23] to the unifactor and the bifactor models [47]. The internal consistency of the scale was calculated by estimating Cronbach’s alpha [48] and McDonald’s omega [49]. Pearson’s *r* correlation was used to investigate the associations between the subscales, as well as with other variables, to assess some aspects of construct validity. Discriminant validity was further investigated utilizing the heterotrait–monotrait ratio of correlations (HTMT) [50] by means of an AMOS plugin [51]. HTMT scores < 0.85 suggested acceptable values [50].

Throughout, statistical significance was set as *p* ≤ 0.05 and was two-tailed.

## 3. Results

The item analysis showed skewness values ranging from −1.197 (item 4) to +0.294 (item 7) and kurtosis values ranging from −1.863 (item 7) to +1.657 (item 4), suggesting a normal distribution. The KMO index of 0.800 and the statistical significance of the Bartlett’s test of sphericity (*p* < 0.001) suggested the suitability of the data for factor analysis.

**Figure 1:**
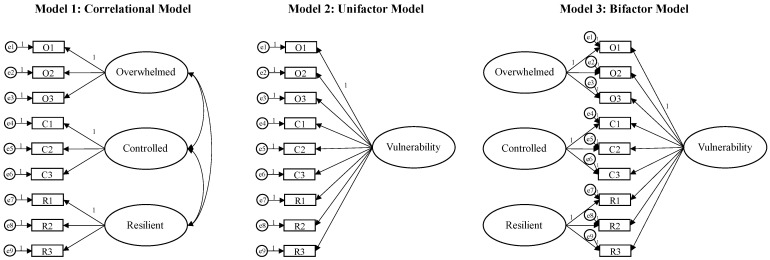
The tested factor structure models for the AAG.

In the CFA, the correlational model showed a good fit: *χ*^2^/df = 3.151; NFI = 0.930; TLI = 0.914; CFI = 0.950; RMSEA = 0.077; and SRMR = 0.056. The chi-square differences (see Table 2) confirmed a statistically significant superior fit of the correlational model, compared with the unifactor model. However, when the correlational model was compared to the bifactor model, the latter showed a statistically significant improvement in the model fit: *χ*^2^/df = 1.810; NFI = 0.979; TLI = 0.968; CFI = 0.990; RMSEA = 0.047; and SRMR = 0.018 (see Figure 1).

Cronbach’s alpha and McDonald’s omega coefficients showed acceptable values for the three subscales (see Table 3). Concerning the total score, the internal reliability indices were poor (*α* = 0.427; *ω* = 0.269) if the scoring of the items suggested in the original English version of the scale was maintained. In contrast, upon reversing the items attributable to a controlled style, the internal reliability indices were significantly better: *α* = 0.746 and *ω* = 0.742. This was further confirmed by the significant and positive association between the controlled and resilient subscales (*r* = 0.567, *p* < 0.001).

Concerning Pearson’s *r* correlation analysis, significant associations emerged between the AAG and the other variables, in terms of both the total score and the subscales (see Table 4). More specifically, the AAG total score was significantly and negatively correlated with the mature defenses (*r* = −0.187; *SE* = 0.051; *p* < 0.001) and positively associated with the immature ones (*r* = 0.110; *SE* = 0.052; *p* < 0.05). Furthermore, the AAG total score showed significant and positive associations with the post-traumatic symptoms, in relation to both the total score (*r* = 0.391; *SE* = 0.048; *p* < 0.001) and the subscales (see Table 4). Additionally, the AAG total score was significantly and positively correlated with depressive symptoms, in relation to both the total score (*r* = 0.435; *SE* = 0.047; *p* < 0.001) and the subscales (see Table 4). These associations provided evidence of construct validity.

Finally, the HTMT analysis showed associations below the threshold value of 0.85, suggesting the absence of problems with discriminant validity for the AAG (see Table 3 and Table 4).

## 4. Discussion

Although many people can be resilient in response to negative and disturbing life events, some individuals may show increased vulnerability to situations of loss and experience significant impairment in their functioning [52,53,54]. Given this, the use of screening tools that evaluate the components of this vulnerability appears to be of great clinical relevance, favoring the development of targeted and effective interventions [55,56]. In line with this, the present research aimed to develop an Italian version of the AAG [22,23] and evaluate its psychometric properties among a sample of adults who lived through the experience of loss.

Results showed that the Italian AAG had satisfactory psychometric properties and provided evidence supporting the internal consistency and construct validity of the measure. Concerning the factor structure, data confirmed the goodness-of-fit of the three-factor correlational solution, which was in line with the original version [22], but it also highlighted that the bifactor model [57,58] provided a superior fit, compared to previously tested models (see Figure 1). This supported the possibility of considering both the general factor as a unitary construct reflecting the common variance across all items (i.e., vulnerability) and the three factors read as conceptually narrower domain constructs (i.e., overwhelmed, controlled, resilient) [59] that were shown to be clearly distinct from each other through HTMT analysis [50]. A further difference from the first version concerned the “controlled” dimension, which was originally conceived as an unreflective/instinctive reaction, directly involved in contributing to vulnerability [22,23]. Instead, the data from the present study suggested the possibility of considering control as a “protective” factor in the Italian population, showing significant and positive associations with the other positive dimension conceived in the scale (i.e., “resilient”), suggesting that one might interpret this dimension as a conscious effort to react and activate one’s resources in response to pain without being overwhelmed by it. In support of this, this factor was negatively correlated with post-traumatic and depressive symptom scores, similar to the “resilient” factor and in line with previous evidence showing the protective role of self-control for mental health [60,61,62]. Furthermore, the “controlled” dimension was significantly and positively related to mature defenses and, to a lesser extent, immature ones. This reflects the idea that, although greater use of mature defenses has been found to be protective for mental health [63,64,65], a functional response to stressful experiences is the result of a balanced integration of different types of mechanisms according to the circumstances [66]. In addition, this structure and relationship between the factors received further support from the Cronbach’s alpha [48] and McDonald’s omega [49] coefficients, which appeared satisfactory for both the subscales and the total score when the “positive” dimensions (i.e., controlled, resilient) were revised, also corroborating the internal consistency of the AAG.

Results showed that the Italian AAG also demonstrated satisfactory performance in terms of some aspects of construct validity; thus, the total score was significantly correlated with the measures used to evaluate convergent validity while maintaining good discriminating power, as evidenced by the HTMT analysis [50]. Specifically, higher vulnerability was associated with higher levels of post-traumatic and depressive symptoms. This echoes previous evidence highlighting that subjects with worse psychological outcomes related to grief have higher levels of depression and PTSD severity, unlike those who have experienced losses but present higher levels of resilience [67,68,69]. Accordingly, the perception of being emotionally overwhelmed in response to a loss may be an important risk factor for negative mental health consequences [70]. Finally, vulnerability levels were significantly and positively associated with the use of immature defenses, in line with previous research showing that the use of these defense mechanisms can lead to a greater impact of negative events [71,72,73]; on the other hand, a significant and negative correlation was shown between vulnerability and mature styles, supporting previous evidence suggesting that the psychological consequences of the event may be influenced by the personal use of defense mechanisms [74].

### Limitations and Suggestions for Future Research

This study had some limitations that should be identified. First, the research involved participants recruited using snowball sampling. Although this method was implemented by including in the research only those who experienced loss, participants were self-selected, and some individuals in this category might not have completed the survey; therefore, this procedure might limit the generalizability of the results. Future research could overcome this limitation by implementing a probability sampling method. In line with the previous point, the original English version of the scale was constructed and validated on subjects recruited into community-based and hospice-based bereavement services and who were either recently bereaved or still actively dealing with the impact of the death of someone close [22,23]. In contrast, the participants in the present research probably considered the loss retrospectively. This aspect could explain the difference in the interpretations of the “controlled” scale that was shown in the two contexts and suggests the need for future research to investigate any variations between the use of the AAG in the immediacy of the loss and its subsequent use when the conscious processes begin to develop as a means of coping, including control. Furthermore, the sample used in this research presented a gender imbalance (78% were female). The replication of the results in a more balanced sample is needed in future research to address this issue. Finally, data were collected by using self-report measures, and this could have led to some biases. The use of a multimethod approach (e.g., by integrating data collection with the use of clinical interviews) could be an important challenge for future research.

## 5. Conclusions

Responses to loss are influenced by individual variability and, in some cases, can lead to significant impairment in the individual’s functioning [75]. With a view to providing a useful tool to contribute to the development of tailored and effective treatments, this research aimed to explore the psychometric properties of the Italian Adult Attitude to Grief scale (AAG; see Appendix A). Results suggested that the AAG is a short (9 items) scale, can quickly be completed, and is simple in its administration and evaluation, which may be effectively used in the Italian context. Evidence was also provided of its good psychometric properties, supporting the possibility of using this self-report tool both for research and clinical practice to easily assess the levels of vulnerability to loss and the constitutive dimensions (overwhelmed, controlled, and resilient) in Italian-speaking populations.

## Figures and Tables

**Table 1:** Demographic features and percentages of loss-experience type in the sample (*n* = 367).

Characteristics		*M* ± *SD*	*n*	%
*Age*		30.44 ± 11.206		
*Sex*				
	Females		285	77.7
	Males		82	22.3
*Marital status*				
	Single		261	71.1
	Married		55	15
	Cohabiting		39	10.6
	Divorced		9	2.5
	Separated		2	0.5
	Widowed		1	0.3
*Education*				
	Elementary school		1	0.3
	Middle School diploma		30	8.2
	High School diploma		123	33.5
	University degree		125	34.1
	Master’s degree		64	17.4
	Post-lauream specialization		24	6.5
*Occupation*				
	Student		134	36.5
	Working-student		55	15
	Employee		85	23.2
	Freelance		30	8.2
	Entrepreneur		11	3
	Manager		5	1.4
	Artisan		7	1.9
	Trader		6	1.6
	Retired		7	1.9
	Unemployed		27	7.4
*Experience of loss*				
	Mourning		219	59.7
	Separation from important people (e.g., friends, family, partner)		114	31.1
	Loss of work, job opportunities, or important occasions.		34	9.3

**Table 2:** Fit statistics of the AAG for the three factor models and the *χ*^2^ difference test (Δ*χ*^2^).

	*χ* ^2^	*df*	*p*	*NFI*	*TLI*	*CFI*	*RMSEA*	*SRMR*	*Model Comparisons*	Δ*χ*^2^	Δ*df*	*p*
Correlational model	66.171	21	<0.001	0.930	0.914	0.950	0.077	0.056				
									-	-	-	-
Unifactor model	236.913	24	<0.001	0.748	0.647	0.765	0.155	0.109				
									M1-M2	170.742	3	<0.001
Bifactor model	19.905	11	<0.05	0.979	0.968	0.990	0.047	0.018				
									M1-M3	46.266	10	<0.001

***Note:*** M1 = correlational model; M2 = unifactor model; M3 = bifactor model.

**Table 3:** Pearson coefficients, standard errors of each Pearson’s *r* (in round brackets), internal reliability indices (α and ω; below the diagonal), and the heterotrait–monotrait (HTMT) correlation ratio for discriminant validity (above the diagonal).

	1	2	3
1. Overwhelmed	—	0.037	0.359
2. Controlled	–0.035 (0.052)	—	0.790
3. Resilient	**–0.267** (0.050)	**0.567** (0.043)	—
*α*	0.701	0.638	0.793
*ω*	0.703	0.643	0.800

***Note:*** Bold values indicate significant indices (both *p* < 0.001).

**Table 4:** Pearson’s correlations and the heterotrait–monotrait (HTMT) correlation ratio for discriminant validity (in round brackets).

		AAG Total Score: Vulnerability	Overwhelmed	Controlled	Resilient
*Defense* *mechanisms*					
	Mature	−0.187 *** (0.386)	−0.008 (0.023)	0.195 *** (0.331)	0.223 *** (0.362)
	Neurotic	0.094 (0.248)	0.224 *** (0.328)	0.080 (0.157)	–0.025 (0.025)
	Immature	0.110 * (0.180)	0.219 *** (0.277)	0.107 * (0.159)	–0.089 (0.090)
*Post-traumatic* *symptoms*					
	Total score	0.391 *** (0.248)	0.336 *** (0.413)	−0.152 ** (0.185)	−0.339 *** (0.393)
	Intrusion	0.289 *** (0.059)	0.266 *** (0.347)	−0.058 (0.069)	−0.278 *** (0.343)
	Avoidance	0.400 *** (0.128)	0.338 *** (0.423)	−0.203 *** (0.255)	−0.312 *** (0.369)
	Hyperarousal	0.374 *** (0.130)	0.310 *** (0.394)	−0.141 ** (0.181)	−0.339 *** (0.406)
*Depressive* *symptoms*					
	Total score	0.435 *** (0.248)	0.298 *** (0.362)	−0.202 *** (0.262)	−0.431 *** (0.500)
	Somatic and Affective	0.388 *** (0.205)	0.277 *** (0.343)	−0.173 *** (0.226)	−0.377 *** (0.444)
	Cognitive	0.436 *** (0.281)	0.285 *** (0.356)	−0.210 *** (0.284)	−0.441 *** (0.528)

***Note***: Bold values indicate significant *p*-values. * *p* < 0.05, ** *p* < 0.01, *** *p* < 0.001.

## Data Availability

The data presented in this study are available upon request from the corresponding author. The data are not publicly available for privacy reasons.

## Appendix A. The Adult Attitude to Grief Scale—Italian Version

Per favore, legga attentamente le seguenti affermazioni e, pensando ad un suo evento di perdita, indichi la risposta che più la rappresenta sulla seguente scala:

**Fortemente D’accordo**	**D’accordo**	**Né D’accordo né in** **Disaccordo**	**In Disaccordo**	**Fortemente in** **Disaccordo**
**4**	**3**	**2**	**1**	**0**

1. Mi sento in grado di affrontare il dolore che deriva dalla perdita.	4	3	2	1	0
2. Per me, è difficile reprimere i pensieri sulla persona che ho perso.	4	3	2	1	0
3. Mi sento molto consapevole della mia forza interiore quando affronto il dolore.	4	3	2	1	0
4. Credo di dover essere coraggioso nell’affrontare la perdita.	4	3	2	1	0
5. Sento che porterò sempre con me il dolore della sofferenza.	4	3	2	1	0
6. Per me, è importante tenere sotto controllo il mio dolore.	4	3	2	1	0
7. La vita ha meno significato per me dopo questa perdita.	4	3	2	1	0
8. Penso che sia meglio andare avanti con la vita e non soffermarsi su questa perdita.	4	3	2	1	0
9. Potrebbe non sempre sembrare così, ma credo che supererò questa esperienza di dolore.	4	3	2	1	0

***Note:*** Translated from the English version © Linda Machin [22,23]; The “*overwhelmed*” dimension can be calculated by summing items 2, 5, and 7. The “*controlled”* dimension can be calculated by summing items 4, 6, and 8. The “*resilient*” dimension can be calculated by summing items 1, 3, and 9. The *vulnerability indicator* (total score) can be obtained by summing all the items after reversing those of the “controlled” and “resilient” dimensions.
